# Recommendations for structural magnetic resonance imaging in infants with first afebrile seizure or new onset epilepsy: Evidence‐based recommendations from the ILAE Neuroimaging Task Force

**DOI:** 10.1002/epi.70205

**Published:** 2026-03-27

**Authors:** Gavin P. Winston, Simone Salemme, Gaetano Cantalupo, Fernando Cendes, Felice D′Arco, Paolo Federico, William D. Gaillard, Eliane Kobayashi, Edward J. Novotny, Godwin Ogbole, Abdul Kareem Pullattayil, Domenico Tortora, Matthew T. Whitehead, Taoyun Yi, Francesco Brigo, Anna Elisabetta Vaudano

**Affiliations:** ^1^ Division of Neurology, Department of Medicine Queen's University Kingston Ontario Canada; ^2^ Centre for Neuroscience Studies Queen's University Kingston Ontario Canada; ^3^ Department of Biomedical, Metabolic, and Neural Sciences University of Modena and Reggio Emilia Modena Italy; ^4^ Department of Engineering for Innovation Medicine University of Verona Verona Italy; ^5^ Child Neuropsychiatry Unit Verona University Hospital, Azienda Ospedaliera Universitaria Integrata Verona Verona Italy; ^6^ Center for Research on Epilepsy in Pediatric Age Azienda Ospedaliera Universitaria Integrata Verona Verona Italy; ^7^ Department of Neurology University of Campinas Campinas Brazil; ^8^ Department of Neuroradiology Great Ormond Street Hospital for Children NHS Foundation Trust London UK; ^9^ Department of Clinical Neurosciences, Cumming School of Medicine University of Calgary Calgary , Alberta Canada; ^10^ Division of Neurology Children's National Hospital, George Washington University Washington District of Columbia USA; ^11^ Department of Neurology, University of Texas Southwestern Medical Center and Peter O'Donnell Jr. Brain Institute Dallas Texas USA; ^12^ Division of Child Neurology, Department of Neurology Seattle Children's Hospital, University of Washington Seattle Washington USA; ^13^ Norcliffe Foundation Center for Integrative Brain Research Seattle Washington USA; ^14^ Department of Radiology, College of Medicine University of Ibadan Ibadan Nigeria; ^15^ Queen's University Library Kingston Ontario Canada; ^16^ Neuroradiology Unit, Department of Services, IRCCS Istituto Giannina Gaslini Genoa Italy; ^17^ Division of Neuroradiology, Department of Radiology Children's Hospital of Philadelphia Philadelphia Pennsylvania USA; ^18^ Department of Radiology Perelman School of Medicine, University of Pennsylvania Philadelphia Pennsylvania USA; ^19^ Department of Pediatrics Peking University First Hospital Beijing China; ^20^ Innovation, Research, and Teaching Service (SABES‐ASDAA), Teaching Hospital of the Paracelsus Medical Private University Bolzano Italy; ^21^ Neurophysiology Unit and Epilepsy Center, Neuroscience Department Modena Azienda Ospedaliera‐Universitaria Modena Italy

**Keywords:** afebrile seizure, epilepsy, infants, MRI, neuroimaging

## Abstract

Infants aged 1–24 months with new onset epilepsy frequently present with structural brain abnormalities, yet no updated evidence‐based magnetic resonance imaging (MRI) guidelines exist for this population. The International League Against Epilepsy (ILAE) Neuroimaging Task Force developed evidence‐based recommendations for structural brain MRI in infants with a first afebrile seizure or new onset epilepsy. A multidisciplinary panel defined three PICO (patients, intervention, comparison group, outcome under consideration) questions, conducted a systematic review (PROSPERO [Prospective Register of Systematic Reviews] CRD42024592653), and reported the results in line with PRISMA (Preferred Reporting Items for Systematic Reviews and Meta‐Analyses) 2020 guidelines. Risk of bias was evaluated using the JBI (Joanna Briggs Institute) checklist. GRADE (Grading of Recommendations, Assessment, Development, and Evaluation) methodology was used to assess certainty of evidence and formulate recommendations for the following: (1) the effectiveness of MRI in identifying underlying etiologies, (2) clinical predictors of MRI abnormalities, and (3) MRI protocols. Seventeen studies (*n* = 1209) were included. Among 753 infants who underwent MRI, 438 (58.2%) had abnormal findings. Despite heterogeneity in MRI protocols and reporting, the evidence supports the utility of MRI in this population. Specific clinical features (focal seizure semiology, abnormal neurological examination, seizure duration > 5 min, focal electroencephalographic abnormalities, developmental delay, and perinatal complications) were associated with abnormal MRI findings, although methodological limitations reduce certainty. Only six studies provided data on MRI sequences; however, none reported findings specifically in relation to the diagnostic accuracy or yield of individual protocols, precluding the development of evidence‐based recommendations on MRI protocol selection. MRI is conditionally recommended in all infants with a first afebrile seizure or new onset epilepsy. MRI could be prioritized in those with specific clinical features indicative of higher likelihood of abnormal findings. Recommendations are based on very low certainty of evidence. These are the first ILAE‐endorsed, evidence‐based recommendations for MRI in infants with first afebrile seizure or new onset epilepsy. Further prospective studies with standardized protocols are needed to refine MRI indications and optimize diagnostic yield in this age group.

**Key points**

- First afebrile seizures or new onset epilepsy in infants are frequently associated with structural brain abnormalities.
- MRI is recommended for infants with first afebrile seizure or new onset epilepsy, as 58% of patients have abnormal findings.
- Clinical features such as focal seizures or EEG abnormalities, abnormal examination, and seizures lasting >5 min increase risk of MRI abnormality.
- Insufficient data exist to provide evidence‐based recommendations on optimal MRI protocol.

## 1 INTRODUCTION

The incidence of epilepsy is highest in infancy,^1^ where it is often associated with structural brain abnormalities, making neuroimaging studies crucial for diagnosis and treatment. Despite differences in brain myelination that affect the choice of magnetic resonance imaging (MRI) sequences, no recent evidence‐based guidelines specifically address this age group.

The International League Against Epilepsy (ILAE) has issued several neuroimaging recommendations. In 1997, MRI was endorsed over computed tomography (CT) in most nonacute situations.^2^ Reflecting significant advances in MRI technology over recent decades, the HARNESS‐MRI (Harmonized Neuroimaging of Epilepsy Structural Sequences) minimum recommended protocol was introduced, comprising high‐resolution three‐dimensional (3D) T1‐weighted and T2 fluid‐attenuated inversion recovery (FLAIR) images and a high in‐plane resolution 2D coronal T2‐weighted image.^3^

This protocol was conceived to be primarily applicable to adults, although the overall principles are generalizable to children older than 24 months. Children younger than 24 months require special sequences, as immature myelination affects the ability to identify common causes of epilepsy. T1‐weighted and T2 FLAIR images may be less useful in infants younger than 24 months due to incomplete myelination, whereas high‐resolution T2‐weighted sequences obtained in 2–3 axes are more important to identify abnormalities including malformations of cortical development/cortical dysplasia. No specific recommendations have been proposed for a population aged less than 24 months.

In 2009, the ILAE Subcommittee for Pediatric Neuroimaging recommended neuroimaging for the evaluation of all children with newly diagnosed epilepsy,^4^ concentrating on CT and 1.5‐T MRI. Indications included focal seizures (based on semiology, examination, or electroencephalography [EEG]), developmental regression, or age less than 24 months. A consensus protocol was suggested with specific recommendations for those younger than 24 months, including a high‐resolution 3D T1‐weighted sequence, T2‐weighted and FLAIR imaging in axial/coronal planes, and oblique coronal T2‐weighted fast spin echo imaging of the hippocampus. High‐resolution T2‐weighted scans in at least two directions were considered particularly important in the first year of life to detect focal cortical dysplasia. Higher field MRI (e.g., 3T) was not addressed. The ILAE Commission on Pediatrics presented a level A recommendation in 2015 for neuroimaging in all infants with epilepsy, ideally MRI, but did not discuss the protocol.^5^

To fill these gaps, the ILAE Neuroimaging Task Force was thus tasked with developing recommendations for the use of structural MRI in infants with unprovoked first seizure or new onset epilepsy focusing on the following: (1) the effectiveness of brain MRI in emergency and nonurgent settings in identifying the etiology; (2) the clinical features associated with presence of MRI‐detected abnormalities; and (3) specifying a minimum recommended MRI protocol tailored to infants. Excluded were infants with provoked seizures (i.e., by fever, infections, trauma, and electrolyte disturbances, transient metabolic or endocrine disorders) and those with seizures confined to the neonatal period (<4 weeks corrected gestational age).

These recommendations focus specifically on the role of MRI and are not intended to compare MRI with other imaging modalities such as CT. They aim to guide clinicians—including pediatric neurologists, epileptologists, neuroradiologists, pediatricians, and emergency care providers—in the diagnostic evaluation of infants with unprovoked seizures across diverse health care settings.

## 2 MATERIALS AND METHODS

### 2.1 Panel composition and consensus process

In September 2022, the ILAE Neuroimaging Task Force convened a dedicated panel composed of seven members from the Neuroimaging Taskforce. The panel included adult epileptologists, pediatric epileptologists, and neuroradiologists from North America, Europe, Latin America, and Africa, representing a diverse range of ILAE regions. To ensure broader expertise and methodological rigor, eight external experts were invited to join the panel from different regions (ILAE North America, ILAE Europe, ILAE Asia, and ILAE Oceania). These included three pediatric epileptologists, three neuroradiologists with specialized knowledge in pediatric neuroimaging, and two methodologists with experience in guideline development and evidence synthesis. All panel members disclosed potential conflicts of interest, which were reviewed and managed in accordance with international best practices. The project was overseen in its main stages by the ILAE Standards and Best Practice Council and followed the reporting guidance outlined in the ILAE‐endorsed toolkit for guideline development and reporting.^6^ Although no formal patient or caregiver representatives were included in the panel, values and preferences were considered based on clinical experience and literature regarding sedation‐related risks, accessibility of pediatric neuroimaging, and family‐centered care in early epilepsy management.

### 2.2 Priority questions

The working group developed a set of priority questions to address in these evidence‐based recommendations. These were drafted using the PICO format (patients, intervention, comparison group, and outcome under consideration) for each question.^7^ These questions and their associated outcomes of interest were used as the basis for a subsequent systematic literature review.

The following PICO questions were formulated:

- PICO 1: What is the effectiveness of brain MRI performed in both emergency and nonurgent settings in identifying the underlying cause of seizures in infants experiencing a first afebrile seizure or new onset epilepsy?
- PICO 2: Which clinical features in infants with a first afebrile seizure or new onset epilepsy are associated with MRI‐detected abnormalities that explain the cause of seizures?
- PICO 3: Which MRI protocols and sequences are most effective in detecting brain abnormalities in infants with a first afebrile seizure or new onset epilepsy?

These questions were developed and refined by a multidisciplinary expert panel comprising pediatric neurologists, epileptologists, neuroradiologists, and methodologists.

### 2.3 Systematic review

We conducted a systematic review, the results of which were reported in accordance with Preferred Reporting Items for Systematic Reviews and Meta‐Analyses (PRISMA) 2020 guidelines.^8^ The review protocol was registered in the Prospective Register of Systematic Reviews (PROSPERO) database (CRD42024592653) under the title “MRI Essentials in Infants With Epilepsy.” We included original studies involving infants (aged 1–24 months) with a first afebrile seizure or new onset epilepsy who underwent brain MRI. Eligible studies had to report at least one of the following: (1) the proportion of patients undergoing MRI, (2) the prevalence and types of MRI abnormalities, and (3) the association between clinical features and MRI findings. Exclusion criteria were studies focusing exclusively on febrile seizures, neonates (<28 days old), or children with a known diagnosis of genetic, metabolic, or structural brain disorders prior to seizure onset. During review, case reports of fewer than five infants and conference abstracts were excluded.

We systematically searched the MEDLINE, Embase, and Web of Science databases from inception to April 13, 2023, using predefined combinations of keywords related to epilepsy, seizures, infants, and MRI. The search strategy was developed in collaboration with a health sciences librarian (A.K.P.). Review articles were collated only to ensure that no key references were missed. The complete search strategy is reported in Tables S1–S3 and corresponds to the strategy preregistered in the PROSPERO protocol.

**FIGURE 1:**
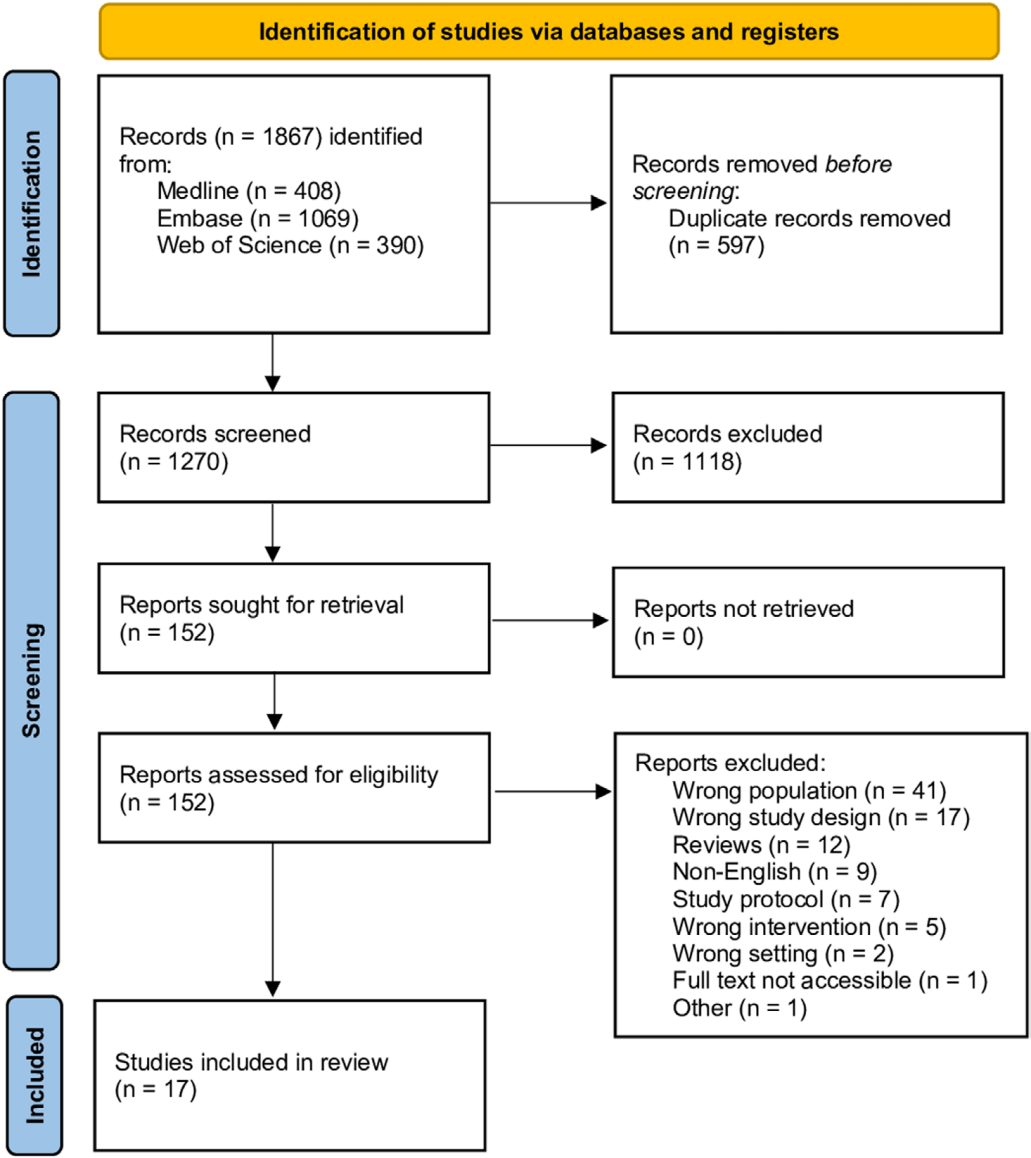
PRISMA (Preferred Reporting Items for Systematic Reviews and Meta‐Analyses) 2020 flow diagram for new systematic reviews that included searches of databases and registers only. *Source*: Page et al.^8^ This work is licensed under CC BY 4.0. To view a copy of this license, visit https://creativecommons.org/licenses/by/4.0/.

All titles and abstracts were independently screened in duplicate by pairs of reviewers using Covidence systematic review software. Full‐text articles deemed potentially eligible were then assessed against predefined inclusion criteria. Discrepancies were resolved through discussion or, when necessary, by consulting a third independent reviewer (G.P.W.). A PRISMA flow diagram summarizing the selection process is available in Figure 1. Data extraction forms for all priority questions were drafted using Covidence software (S.S.) and pilot‐tested by selected members of the working group (G.P.W., A.E.V.).

Extracted variables included the following: (1) general study characteristics (e.g., design, country, sample size, setting), (2) population details (age, sex distribution, inclusion/exclusion criteria), (3) clinical features (seizure semiology, neurological exam, EEG findings, perinatal complications, developmental delay), (4) MRI parameters (protocol, sequences, timing, scanner type), (5) MRI outcomes (proportion and type of abnormalities), and (6) predictors of abnormal MRI. When available, (7) therapeutic outcomes related to MRI findings, such as changes in medical or surgical management, were also collected. Evidence synthesis tables were developed for each PICO question, summarizing study characteristics, key findings, and quality assessments (see Tables S4–S6).

### 2.4 Risk of bias and certainty of evidence assessment

Studies meeting inclusion criteria and considered relevant to a priority question were evaluated. Risk of bias was independently assessed by a member of the working group (S.S.) using the JBI (Joanna Briggs Institute) Critical Appraisal Checklist for Analytical Cross‐Sectional Studies.^9^ This tool was chosen in place of the originally planned ROBINS‐I^10^ due to the predominance of cross‐sectional observational designs among the included studies. In parallel, studies were also classified according to the Classification of Evidence framework from the 2017 American Academy of Neurology Clinical Practice Guideline Process Manual.^11^

### 2.5 Formulation of recommendations

Grading of Recommendations, Assessment, Development, and Evaluation (GRADE) was applied following internationally accepted standards for the development of evidence‐based recommendations.^12^ For each PICO question, the certainty of the body of evidence was assessed across the five GRADE domains: (1) risk of bias, (2) inconsistency, (3) indirectness, (4) imprecision, and (5) publication bias. Each domain was rated as “not serious,” “serious,” or “very serious.” The rationale for each rating is presented in the evidence profiles and Summary of Findings tables, developed separately for each PICO question (see Tables S7 and S8).

The recommendation process was conducted through virtual meetings held between September 2022 and September 2024, over five sessions. Consensus on judgments and recommendations was reached through structured discussion, without the need for formal voting procedures. When differing views emerged, further dialogue was facilitated by the methodological team to achieve agreement.

Recommendations were developed through a structured process that considered the following: (1) the balance between desirable and undesirable effects; (2) the certainty of evidence; (3) stakeholder values and preferences; and (4) feasibility and resource implications, such as MRI availability and the need for sedation in infants. Each recommendation was classified as (1) strong, if the panel judged that benefits clearly outweighed harms (or vice versa) for most patients; or (2) conditional, if there was uncertainty or variability in the balance of effects, values, or resource use, or if evidence was limited. GRADE Evidence‐to‐Decision frameworks were used to guide judgments in a transparent and systematic way.

## 3 RESULTS

### 3.1 Study selection

The literature search yielded a total of 1867 records. After removing duplicates, 1270 titles and abstracts were screened. Of these, 152 full‐text articles were assessed for eligibility, and 17 studies were included in the final analysis. The selection process is illustrated in the PRISMA flow diagram (Figure 1).

### 3.2 Study characteristics

The included studies comprised a total of 1209 infants aged 1–24 months with a first afebrile seizure or new onset epilepsy. Of these, 753 infants (62.3%) underwent brain MRI. The studies were heterogeneous in terms of study design, clinical setting, MRI protocols, and population characteristics. MRI utilization rates varied substantially, from 49.1%^13^ to 100%.^14^, ^15^, ^16^, ^17^ Notably, most studies enrolled pediatric populations extending beyond infancy; in these cases, only data pertaining to children under 24 months were extracted and analyzed as far as possible (Table 1).

**TABLE 1:** Included studies.

Study ID	Country	Inclusion criteria	Infants total, *n*
Al‐Shami 2016	QAT	Age: <14 years; first afebrile seizure	26 (<2 years)
Ali 2022	PAK	Age: 1 month–18 years; new onset afebrile seizures	83 (<1 year)
Aprahamian 2014	USA	Age: 1 month–18 years; first time nonfebrile seizure with focal manifestations	72 (<18 months)
Berg 2000	USA	Age: 1 month–15 years; new diagnosis of epilepsy	N.R.
Berg 2009	USA	Age: 1 month–16 years; new diagnosis of epilepsy	N.R.
Cornelius 2023	IND	Age: <12 years; new onset seizures (due to inherited metabolic disorder)	32 (<1 year)
Coryell 2018	USA	Newly diagnosed early life epilepsy; first seizures <3 year; established epilepsy diagnosis at <42 months	N.R.
Dirik 2018	TUR	Age: 1–18 years; new diagnosis of epilepsy	N.R.
Eltze 2013	GBR	Age: 1–24 months; new diagnosis of epilepsy	57
Gattamaneni 2022	IND	Age: 1 month–5 years; new onset seizures^a^	46
Gowda 2019	IND	Age: 1 month–1 year; first afebrile seizure	121
Hourani 2021	LBN	Age: 6 months–18 years; new onset unprovoked seizure(s)	169 (<2 years)
Hsieh 2010	USA	Age: 1–24 months; new onset afebrile seizures	317
Kasap 2023	TUR	Age: 1 month–18 years; first focal seizure^a^	15 (<1 year)
Stödberg 2020	SWE	New diagnosis of epilepsy with first seizure at <2 years	116
Trowbridge 2019	USA	Down syndrome + infantile spasms who had MRI	36
Vecchi 2016	ITA	Age: 1 month–13 years; diagnosis of symptomatic epilepsy due to acquired and developmental etiologies and presumed symptomatic focal epilepsy	119 (<3 years)

*Note*: Country codes refer to the country of origin of each study, based on ISO 3166‐1 alpha‐3 codes. Abbreviations: GBR, United Kingdom; IND, India; ITA, Italy; LBN, Lebanon; MRI, magnetic resonance imaging; N.R., not reported; PAK, Pakistan; QAT, Qatar; TUR, Turkey; SWE, Sweden; USA, United States.

^a^ A proportion of the infants in this study had a febrile seizure etiology; however, the study was retained as it also includes infants within the target population of interest (first afebrile seizure or new‐onset epilepsy).

Incompleteness in reporting was common; four studies did not report the total number of infants evaluated,^18^, ^19^, ^20^, ^21^ nine studies lacked data on MRI utilization,^14^, ^19^, ^20^, ^21^, ^22^, ^23^, ^24^, ^25^, ^26^ and eight studies did not specify the number of abnormal MRI findings.^19^, ^20^, ^21^, ^22^, ^23^, ^24^, ^26^, ^27^

These gaps limit the precision of aggregated estimates and may contribute to the underestimation of diagnostic yield. A detailed overview of study characteristics is provided in Tables S1 and S5. A quantitative synthesis (meta‐analysis) was not performed due to substantial clinical and methodological heterogeneity, including variability in populations, definitions of clinical features, MRI protocols, and outcome measures. No patient‐reported outcomes were reported in any of the included studies.

### 3.3 Recommendation #1: Diagnostic yield of brain MRI

#### 3.3.1 Evidence synthesis

Among the infants undergoing MRI, 438 (58.2%) were reported to have abnormal neuroimaging findings. The proportion of abnormal scans ranged from 48.5%^15^ to 72.5%,^28^ reflecting heterogeneity in case selection, MRI timing, and neuroimaging protocols. Whereas some studies reported a high diagnostic yield,^16^, ^28^, ^29^ others lacked clarity regarding the clinical relevance of MRI findings. A detailed overview of study characteristics is provided in Table S4, and a summary of findings with certainty assessment is provided in Table S7.

#### 3.3.2 GRADE assessment

**Table:** 

GRADE domain	Evaluation	Rationale
Risk of bias	Not serious	Several studies have incomplete reporting on infant numbers, MRI use, and detected abnormalities. However, no major methodological flaws are suspected, and the data remain a reasonable basis for assessing MRI effectiveness.
Inconsistency	Not serious	Although the proportion of infants undergoing MRI and the rate of detected abnormalities vary widely—indicating heterogeneity in study populations, imaging protocols, and reporting practices—this variability is likely attributable to differences in inclusion/exclusion criteria, clinical settings, and technical MRI parameters.
Indirectness	Very serious	Variability in MRI protocols, inclusion criteria, and the classification of abnormalities limits the direct applicability of findings to all clinical settings. Some studies do not distinguish between clinically relevant and incidental findings.
Imprecision	Serious	Confidence in effect estimates is limited by incomplete data reporting, wide variability in diagnostic yield across studies, and differences in neuroradiologists' expertise. Although some findings are based on small sample sizes, substantial variability is also observed within these cohorts.
Publication bias	Not serious	The presence of unpublished negative studies cannot be ruled out, but no strong indication of publication bias is evident from the data provided.

Despite multiple limitations, the evidence consistently suggests that brain MRI can identify potentially relevant structural abnormalities in a substantial proportion of infants with new onset seizures.

#### 3.3.3 Evidence‐based recommendation

**Table:** 

**Recommendation** In infants presenting with a first afebrile seizure or new onset epilepsy, brain MRI is conditionally recommended, in either an emergency or nonurgent setting, to identify underlying structural causes. **Quality of evidence**: Very low **Strength of recommendation**: Conditional

##### Rationale

The GRADE assessment rated the overall certainty of evidence as very low. Despite these limitations, the evidence consistently suggests that brain MRI can identify potentially relevant structural abnormalities in a substantial proportion of infants with new onset seizures. This recommendation is conditional, and clinical decision‐making should be guided by local resource availability, patient risk stratification, and the potential impact of MRI findings on management. Clinicians should also consider the risks of imaging, need for sedation, and availability of pediatric imaging expertise. In acute presentations, particularly where MRI is not immediately available or sedation is not feasible, head CT may be used to exclude urgent symptomatic causes that require intervention such as subdural hemorrhage or acute hydrocephalus. However, it should not replace MRI when indicated and available, especially if structural etiology remains suspected.

##### Implications for research

The working panel recommends that future studies focus specifically on infants less than 24 months of age and directly compare the diagnostic yield and clinical impact of brain MRI in this population. Well‐designed comparative effectiveness or prospective studies are warranted to assess whether early MRI influences acute management and long‐term prognosis in this age group.

### 3.4 Recommendation #2: Clinical predictors of MRI abnormalities

#### 3.4.1 Evidence synthesis

Among the 1209 infants included across studies, 371 of 935 infants (39.7%, or 30.7% of total sample) for whom information was available had an abnormal neurological examination, with prevalence ranging from 10.7%^29^ to 100%^16^; 318 of 434 infants (73.3%, or 26.3% of total sample) had abnormal EEG findings, all described as focal, although the nature and localization of EEG alterations were often poorly specified; 94 of 554 infants (17.0%, or 7.8% of total sample) had a history of abnormal pregnancy or delivery or perinatal complications, as reported in the few studies that included this information.^13^, ^25^, ^29^ A detailed overview of study characteristics is provided in Table S5, and a summary of findings with certainty assessment is provided in Table S8.

The most frequently identified clinical features associated with an increased likelihood of abnormal MRI findings included (1) focal seizure semiology, (2) prolonged seizure duration (>5 min), (3) abnormal neurological examination, (4) focal EEG abnormalities, (5) developmental delay, and (6) history of perinatal complications. However, the strength of these associations is limited by incomplete adjustment for confounding variables and inconsistency in how predictors were defined, measured, and reported across studies.

#### 3.4.2 GRADE assessment

**Table:** 

GRADE domain	Evaluation	Rationale
Risk of bias	Serious	Many studies lack complete reporting on the total number of infants evaluated, the presence of abnormal neurological examinations, EEG findings, and perinatal complications. This increases the risk of selection and reporting bias, potentially skewing the associations between clinical features and MRI abnormalities.
Inconsistency	Not serious	The prevalence of abnormal neurological exams, EEG findings, and perinatal complications varies widely, reflecting relevant heterogeneity in study populations, clinical assessments, and reporting practices. However, despite these limitations, no major methodological flaws are suspected, and the findings consistently trend in the same direction, reinforcing their overall validity.
Indirectness	Very serious	Variability in the definitions of clinical features (e.g., focal vs. generalized seizures, abnormal neurological exams), inconsistencies in reporting of EEG interpretations and abnormal neurological examination, and the lack of standardized population descriptions limit the direct applicability of findings. Additionally, some studies do not account for confounders that may influence MRI findings, further reducing the strength of the associations observed.
Imprecision	Serious	The limited sample sizes, along with missing, nonstandardized data on clinical features and reporting inconsistencies, reduce confidence in the precision of effect estimates.
Publication bias	Not serious	The presence of unpublished negative studies cannot be ruled out, but no strong indication of publication bias is evident from the data provided.

Although trends across studies suggest certain clinical features may be associated with abnormal MRI findings, methodological limitations undermine the strength and applicability of these associations.

#### 3.4.3 Evidence‐based recommendation

**Table:** 

**Recommendation** For infants less than 24 months of age with a first afebrile seizure or new onset epilepsy, those exhibiting focal seizure semiology, abnormal neurological examination, prolonged seizure duration (>5 min), focal EEG abnormalities, a history of developmental delay, and perinatal complications could be prioritized for brain MRI, as these may indicate a higher likelihood of detecting an underlying cause. **Quality of evidence**: Very low **Strength of recommendation**: Conditional

##### Rationale

The GRADE assessment rated the overall certainty of evidence as very low. The most frequently identified features associated with abnormal MRI findings included focal seizure semiology, prolonged seizure duration, abnormal neurological examination, focal EEG abnormalities, developmental delay, and perinatal complications. Although the trends across studies suggest that these clinical features may predict abnormal MRI findings, the strength of the association is limited by methodological variability. This recommendation is conditional and should be applied within the context of local imaging availability, diagnostic resources, and clinical judgment.

##### Implications for research

Future studies should implement prospective designs with standardized and comprehensive data collection protocols, including systematic recording of neurological examination findings, detailed seizure semiology, EEG abnormalities, family history, developmental delay, and relevant perinatal or pregnancy complications. Additional research should investigate the impact of MRI timing in relation to dynamic clinical features, such as seizure evolution, postictal findings, and progression of neurological abnormalities. Standardizing the assessment of when MRI is performed relative to these factors may improve understanding of its diagnostic yield and clinical utility.

### 3.5 MRI protocols or sequences associated with detection of abnormalities

Only six studies^15^, ^16^, ^18^, ^20^, ^21^, ^29^ provided partial or complete information on the MRI protocols or sequences used in infants with a first afebrile seizure or new onset epilepsy. However, the reporting was highly heterogeneous, and most studies did not allow for a direct comparison between protocols or for quantification of diagnostic yield stratified by sequence type. Due to the lack of standardized reporting and absence of comparisons, it was not possible to conduct a GRADE assessment or to formulate an evidence‐based recommendation.

Consensus‐based recommendations informed by a systematic synthesis of the available evidence are needed to determine which MRI protocols or sequences are most effective in detecting clinically relevant brain abnormalities in this population. Such recommendations will be developed following a modified Delphi process and provided in a later report.

## 4 DISCUSSION

Epilepsy with onset in the first 2 years of life presents a distinct diagnostic and therapeutic challenge.^13^ Early life seizures are frequently associated with underlying structural abnormalities,^13^ and timely identification of these abnormalities has direct implications for clinical management, prognosis, and, in some cases, treatment selection.^14^, ^20^, ^29^ In those infants with a single afebrile seizure, detection of a lesion associated with high risk of recurrence can lead to a diagnosis of epilepsy, which impacts clinical management. In this context, structural brain imaging—particularly MRI—plays a crucial role in etiological evaluation.

However, prior to this effort, there were no infant‐specific, evidence‐based recommendations to guide clinicians on when and how brain MRI should be used in infants presenting with a first afebrile seizure or new onset epilepsy. This work addresses that gap by formulating recommendations grounded in a systematic synthesis of the best available evidence, following the GRADE methodology, and refined through expert consensus.

### 4.1 Need for infant‐specific recommendations

Although neuroimaging is widely accepted as an essential part of epilepsy evaluation, infants present unique physiological and developmental characteristics that complicate both image acquisition and interpretation. Existing imaging guidelines are typically developed for broader pediatric and/or adult populations and do not specifically account for the unique considerations in children younger than 24 months. For example, the widely endorsed HARNESS‐MRI protocol^3^ has led to improved detection of epileptogenic lesions in older children and adults with focal epilepsy.^30^, ^31^, ^32^ However, it was developed for adults and thus may not be suitable for infants due to developmental differences such as incomplete myelination.

In neonates and young infants, certain sequences key to this protocol such as volumetric T1‐weighted and T2 FLAIR may have limited utility, whereas high‐resolution T2‐weighted imaging is better suited for identifying focal cortical dysplasia and migrational anomalies.^4^, ^33^ Optimal early imaging is critical, as focal cortical dysplasia lesions may become less conspicuous with maturation of myelination.^34^ These age‐specific considerations underscore the need for clinical guidelines, including tailored imaging protocols specifically designed for this developmental window.

### 4.2 Diagnostic utility of MRI in infants

Whether MRI should be performed in all infants presenting with a first afebrile seizure or new onset epilepsy has been a matter of clinical debate. On one hand, early seizures in this age group may reflect underlying structural abnormalities, and timely identification through MRI can accelerate diagnosis, prompt additional investigations (e.g., genetic or metabolic testing), inform prognosis, and even lead to early surgical consideration when appropriate. On the other hand, the universal application of MRI in this population carries significant costs, logistical demands, and potential risks, particularly those associated with sedation in infants.

In this context, the findings of this systematic review and expert consensus offer strong support for the diagnostic utility of MRI in this population of interest. Among the 753 infants who underwent MRI across the 17 included studies, 58.2% were reported to have abnormal neuroimaging findings. This diagnostic yield is higher than that typically reported in older pediatric epilepsy cohorts, reinforcing the value of MRI in early onset cases. However, the range of abnormality rates across studies was wide—from 48.5% to 72.5%—highlighting the underlying heterogeneity in patient selection, MRI protocols, and interpretation criteria.

A major contributor to this heterogeneity is the scarcity of studies focused exclusively on infants. In most cases, data were extracted from broader pediatric cohorts that included children well beyond infancy (e.g., up to 18 years old), with infant‐specific results often not stratified or clearly reported. In some cases, data could only be extracted for those younger than 12 months,^23^, ^27^ 18 months,^22^ or 36 months,^17^ rather than our target population of less than 24 months of age. Although we extracted age‐relevant data where possible, the lack of dedicated infant‐focused studies remains a significant gap in the literature and limits the precision and generalizability of the findings.

Equally important is the limited distinction made in many studies between clinically significant abnormalities—such as malformations of cortical development, hypoxic–ischemic injury, or tumors—and incidental findings of unclear relevance. Studies undertaken in the emergency setting typically refer only to findings resulting in immediate changes in clinical management,^14^, ^22^, ^26^, ^27^ including hemorrhage, infarcts, and tumors, which are typically identified on CT.

Studies outside the emergency setting refer only to findings of etiological relevance,^13^, ^15^, ^18^, ^19^ separately report clinically relevant and incidental findings,^16^, ^20^, ^23^, ^28^, ^29^ or combine both clinically relevant and incidental findings.^17^, ^21^ Common findings include malformations of cortical development, tuberous sclerosis, hypoxic–ischemic injury, tumors, and metabolic disorders. However, the inclusion of incidental findings such as brain atrophy or ventricular dilatation may inflate some of the estimates for the prevalence of abnormalities. Overall, this hampers the interpretability of the data and reduces confidence in the impact of MRI results on clinical management.

Despite these limitations, the consensus panel recommends MRI in all infants with afebrile first seizure or new onset seizures. This recommendation is grounded in the high likelihood of detecting structural pathology, even in the absence of focal neurological signs or clear etiological indicators. However, it is classified as conditional given the very low certainty of evidence, reflecting methodological heterogeneity, incomplete reporting, and a lack of outcome standardization. Clinical decision‐making should weigh the expected diagnostic yield against factors such as the need for sedation, institutional imaging capacity, and availability of pediatric neuroradiological expertise.

It is important to distinguish between infants presenting after a single unprovoked seizure and those with established epilepsy. Although both groups are included in the target population, their clinical trajectories, risk profiles, and imaging priorities may differ. In particular, early MRI may be prioritized in infants with epilepsy, focal neurological signs, or developmental delay.

### 4.3 Feasibility of access to MRI

MRI in infants younger than 2 years often requires sedation or general anesthesia to ensure adequate image quality. Although procedural sedation is generally considered safe when performed by trained personnel under appropriate monitoring, its safety and feasibility may vary depending on the clinical setting, patient age, and comorbidities. These factors should be carefully considered when determining the timing and setting of MRI acquisition. This consideration was, however, not formally addressed in the papers forming our evidence base.

Furthermore, these recommendations are based on evidence specifically for the use of MRI rather than CT and are derived predominantly from studies conducted in high‐resource settings, where MRI access and pediatric radiology expertise are generally available. However, in low‐resource settings, limited access to MRI and sedation may restrict feasibility. In such contexts, head CT—despite its lower sensitivity for cortical malformations or subtle abnormalities—may serve as a complementary tool, particularly in acute settings where rapid exclusion of symptomatic causes is necessary. Decisions regarding neuroimaging should be adapted to clinical urgency and local resource availability.

### 4.4 Clinical features predictive of MRI abnormalities

Another critical question addressed by this review concerns the ability of clinical features to predict which infants are most likely to have abnormal MRI findings. In the emergency setting, clinically significant imaging abnormalities are more common in younger age groups, including less than 18 months,^22^ less than 24 months,^14^ or 1–5 years.^27^ Other predictors include seizure duration greater than 5 min,^14^, ^27^ focal seizures,^27^ focal neurological deficit,^22^, ^27^ or recurrent seizures,^26^ but these were assessed across the whole cohorts, not specifically in infants.

Outside the emergency setting, the strong methodological study designed to address the lack of evidence to guide prior American Academy of Neurology, Child Neurology Society, and American Epilepsy Society guidelines on evaluating a first nonfebrile seizure in children^35^ was a prospective study of 1000 children with new onset unprovoked seizure undergoing MRI.^15^ This confirmed the strong age dependence, with the prevalence of epileptogenic abnormalities being 48.5% in the less than 24‐month age group, which falls to 13.5% by the age of 15–18 years. Imaging abnormalities were more common in association with developmental delay (65% vs. 23% specifically in the less than 24‐month age group), recurrent seizures (across all ages), and seizure type (66% in spasms/tonic/atonic seizures, and 31% in focal seizures across all ages).

Other studies in this setting report associations with developmental delay of cognitive impairment,^17^, ^18^, ^20^, ^29^ abnormal EEG,^19^, ^29^ abnormal examination,^18^, ^19^ spasms^20^ or focal seizures,^19^, ^20^ pharmacoresistance^16^, ^18^ or polytherapy^16^, ^17^ and younger age,^20^ but these are across all age groups addressed in the individual studies.

Overall, these features may assist in stratifying patients by imaging priority, especially in resource‐limited settings where routine MRI for all infants may not be feasible. However, the strength and specificity of these predictors in infants remain uncertain due to methodological limitations in the literature. Although many studies describe some clinical characteristics of the cohort, few studies address whether they are predictive of or associated with MRI abnormalities. When assessed, the predictors are described across the whole cohort of children being studied and are not specific to infants.

Furthermore, definitions of clinical features varied widely across studies. For example, the criteria for “abnormal neurological examination” were inconsistently applied and often conflated with developmental disability; EEG findings were often poorly described in terms of localization, pattern, or timing; and the definition of pregnancy/birth complications ranged from significant history of prenatal insult^25^ to premature birth^29^ to premature or low birthweight.^13^ Finally, few studies adjusted for potential confounders such as seizure type, family history, or timing of MRI relative to seizure onset.

Despite these challenges, the consistency of associations across multiple studies supports the utility of these features in guiding imaging decisions. Accordingly, the panel conditionally recommends prioritizing MRI for infants with these clinical features, even when immediate imaging for all patients is not feasible.

Future research should aim to clarify the predictive value of these features using standardized definitions and prospective data collection. Large, multicenter studies incorporating detailed clinical phenotyping, EEG analysis, and standardized MRI protocols are especially needed to refine risk stratification tools and imaging guidelines in this vulnerable population.

### 4.5 Gaps in evidence and research priorities

Perhaps the most striking finding of this review is not what was present in the literature, but what was absent. There is striking heterogeneity between the included studies in terms of the populations, setting, and data reported. Of the 17 studies, only five focus solely on infants,^13^, ^20^, ^25^, ^28^, ^29^ and subgroup analyses in the other studies and relevant clinical factors such as neurological examination, EEG findings, seizure type, and pregnancy complications are rarely reported. Although the largest study^15^ did provide some subgroup analyses of those aged less than 24 months, only two studies specifically address clinical predictors in this age group alone.^13^, ^20^

The outcome of imaging and definition of abnormality vary, as some studies include both CT and MRI, and whether incidental findings are included is variable. The clinical impact is rarely assessed apart from studies in the emergency department setting. None of the studies conducted formal comparative analyses of MRI protocols. This lack of standardization precluded analysis of which MRI techniques offer the greatest diagnostic yield in infants, an important gap given that the utility of specific sequences (e.g., T2 FLAIR vs. high‐resolution T2‐weighted) differs markedly in this age group due to the ongoing process of myelination.

Thus, although the present recommendations provide a foundation for clinical practice, they also highlight substantial areas in need of further research. There is an especially great need for well‐conducted prospective studies specifically looking at the younger than 24‐month age group with systematically collected data and assessment of imaging outcomes and clinical decision‐making. Given the limited and heterogeneous nature of the available evidence, clinicians are encouraged to apply these recommendations flexibly, considering local diagnostic pathways, imaging access, and system‐level constraints.

## 5 CONCLUSIONS

This work delivers the first comprehensive, evidence‐based recommendations for brain MRI in the context of first afebrile seizure and new onset epilepsy specifically addressing infants aged less than 24 months.

The data demonstrate a high prevalence of actionable structural abnormalities, but significant heterogeneity and imprecision in studies lead to a conditional recommendation for MRI in this population. However, the data are insufficient to recommend an optimal MRI protocol.

Patients with high‐risk clinical features including focal seizure semiology, abnormal neurological examination, prolonged seizure duration (>5 min), focal EEG abnormalities, a history of developmental delay, and perinatal complications could be prioritized, as these are associated with higher risk of MRI abnormalities.

These recommendations are based on very low certainty evidence and are intended to support, rather than dictate, clinical decision‐making. Implementation should consider the local health care context, resource availability, and diagnostic infrastructure. As new evidence emerges, these recommendations should be updated to reflect evolving best practices.

## AUTHOR CONTRIBUTIONS

**Gavin P. Winston:** Conceptualization (supporting); formal analysis; investigation; project administration; writing—original draft preparation (lead). **Simone Salemme:** Formal analysis; investigation; methodology; writing—original draft preparation (supporting). **Gaetano Cantalupo:** Investigation; writing—review and editing. **Fernando Cendes:** Investigation; writing—review and editing. **Felice D'Arco:** Investigation; writing—review and editing. **Paolo Federico:** Investigation; writing—review and editing. **William D. Gaillard:** Investigation; writing—review and editing. **Eliane Kobayashi:** Investigation; writing—review and editing. **Edward J. Novotny:** Investigation; writing—review and editing. **Godwin Ogbole:** Investigation; writing—review and editing. **Adbul Kareem Pullattayil:** Investigation; methodology; writing—review and editing. **Domenico Tortora:** Investigation; writing—review and editing. **Matthew T. Whitehead:** Investigation; writing—review and editing. **Taoyun Yi:** Investigation; writing—review and editing. **Francesco Brigo:** Investigation; methodology; writing—review and editing. **Anna E. Vaudano:** Conceptualization (lead); formal analysis; funding acquisition; investigation; project administration; writing—original draft preparation (supporting).

## CONFLICT OF INTEREST STATEMENT

F.C. has received speaker honoraria and scientific advisory board fees from UCB Pharma, Eurofarma, Libbs, Torrent, Ache, Medley, and Biocodex, and institutional grants from the São Paulo Research Foundation (FAPESP), grant #2013/07559‐3, and CNPq (Conselho Nacional de Desenvolvimento Científico e Tecnológico, Brazil). He is the Editor‐in‐Chief of *Epilepsia*. E.K. receives authorship honoraria for *MedLink Neurology*. E.J.N reports grant support or consultancy fees from UCB, Longboard Pharmaceuticals, LivaNova, Xenon, Cadwell, and Cadence. A.E.V. has received institutional grants from the Italian League Against Epilepsy, Ministry of Health (PNRR‐MR1‐2022‐12 376 491; PRUA1GR‐2013‐00000120), Italian Ministry of Research and University (20229PLPZJ), and Jazz Pharmaceutical. None of the other authors has any conflict of interest to disclose. We confirm that we have read the Journal's position on issues involved in ethical publication and affirm that this report is consistent with those guidelines.

## Supporting information


DATA S1.

## Data Availability

All data are available in the original papers cited and in the evidence summary tables in this paper.
