# Supplementary material for: Recommendations for structural magnetic resonance imaging in infants with first afebrile seizure or new onset epilepsy: Evidence‐based recommendations from the ILAE Neuroimaging Task Force
Source: Epilepsia. 2026 Mar 27;67(6):2725–38. doi: 10.1002/epi.70205 (PMC13285247; doi:10.1002/epi.70205)
Supplement: Supplementary file 1 — DATA S1. [file EPI-67-2725-s001.docx]

**Recommendations for structural magnetic resonance imaging in infants with first afebrile seizure or new onset epilepsy: evidence-based recommendations from the ILAE Neuroimaging Task Force (Supplementary Materials)**

**PRISMA 2020 Checklist**

| **Section and Topic** | **Item #** | **Checklist item** | **Location where item is reported** |
| --- | --- | --- | --- |
| **TITLE** | | |  |
| Title | 1 | Identify the report as a systematic review. | Pg 1 |
| **ABSTRACT** | | |  |
| Abstract | 2 | See the PRISMA 2020 for Abstracts checklist. | Pg 2 |
| **INTRODUCTION** | | |  |
| Rationale | 3 | Describe the rationale for the review in the context of existing knowledge. | Pg 3 |
| Objectives | 4 | Provide an explicit statement of the objective(s) or question(s) the review addresses. | Pg 3,4 |
| **METHODS** | | |  |
| Eligibility criteria | 5 | Specify the inclusion and exclusion criteria for the review and how studies were grouped for the syntheses. | Pg 4,5 |
| Information sources | 6 | Specify all databases, registers, websites, organisations, reference lists and other sources searched or consulted to identify studies. Specify the date when each source was last searched or consulted. | Pg 5 |
| Search strategy | 7 | Present the full search strategies for all databases, registers and websites, including any filters and limits used. | Pg 4, Suppl. Tables 1-3 |
| Selection process | 8 | Specify the methods used to decide whether a study met the inclusion criteria of the review, including how many reviewers screened each record and each report retrieved, whether they worked independently, and if applicable, details of automation tools used in the process. | Pg 5 |
| Data collection process | 9 | Specify the methods used to collect data from reports, including how many reviewers collected data from each report, whether they worked independently, any processes for obtaining or confirming data from study investigators, and if applicable, details of automation tools used in the process. | Pg 5 |
| Data items | 10a | List and define all outcomes for which data were sought. Specify whether all results that were compatible with each outcome domain in each study were sought (e.g. for all measures, time points, analyses), and if not, the methods used to decide which results to collect. | Pg 5 |
|  | 10b | List and define all other variables for which data were sought (e.g. participant and intervention characteristics, funding sources). Describe any assumptions made about any missing or unclear information. | Pg 5 |
| Study risk of bias assessment | 11 | Specify the methods used to assess risk of bias in the included studies, including details of the tool(s) used, how many reviewers assessed each study and whether they worked independently, and if applicable, details of automation tools used in the process. | Pg 5,6 |
| Effect measures | 12 | Specify for each outcome the effect measure(s) (e.g. risk ratio, mean difference) used in the synthesis or presentation of results. | N.A. |
| Synthesis methods | 13a | Describe the processes used to decide which studies were eligible for each synthesis (e.g. tabulating the study intervention characteristics and comparing against the planned groups for each synthesis (item #5)). | Pg 5,6 |
|  | 13b | Describe any methods required to prepare the data for presentation or synthesis, such as handling of missing summary statistics, or data conversions. | Pg 5,6 |
|  | 13c | Describe any methods used to tabulate or visually display results of individual studies and syntheses. | Pg 5,6 |
|  | 13d | Describe any methods used to synthesize results and provide a rationale for the choice(s). If meta-analysis was performed, describe the model(s), method(s) to identify the presence and extent of statistical heterogeneity, and software package(s) used. | Pg 5,6 |
|  | 13e | Describe any methods used to explore possible causes of heterogeneity among study results (e.g. subgroup analysis, meta-regression). | N.A. |
|  | 13f | Describe any sensitivity analyses conducted to assess robustness of the synthesized results. | N.A. |
| Reporting bias assessment | 14 | Describe any methods used to assess risk of bias due to missing results in a synthesis (arising from reporting biases). | Pg 6 |
| Certainty assessment | 15 | Describe any methods used to assess certainty (or confidence) in the body of evidence for an outcome. | Pg 5,6 |
| **RESULTS** | | |  |
| Study selection | 16a | Describe the results of the search and selection process, from the number of records identified in the search to the number of studies included in the review, ideally using a flow diagram. | Pg 7 |
|  | 16b | Cite studies that might appear to meet the inclusion criteria, but which were excluded, and explain why they were excluded. | Fig 1 |
| Study characteristics | 17 | Cite each included study and present its characteristics. | Pg 7, Table 1 |
| Risk of bias in studies | 18 | Present assessments of risk of bias for each included study. | Suppl. Table 6 |
| Results of individual studies | 19 | For all outcomes, present, for each study: (a) summary statistics for each group (where appropriate) and (b) an effect estimate and its precision (e.g. confidence/credible interval), ideally using structured tables or plots. | Pg 7-11, Table 1, Suppl. Tables 7,8 |
| Results of syntheses | 20a | For each synthesis, briefly summarise the characteristics and risk of bias among contributing studies. | Pg 7-11 |
|  | 20b | Present results of all statistical syntheses conducted. If meta-analysis was done, present for each the summary estimate and its precision (e.g. confidence/credible interval) and measures of statistical heterogeneity. If comparing groups, describe the direction of the effect. | N.A. |
|  | 20c | Present results of all investigations of possible causes of heterogeneity among study results. | N.A. |
|  | 20d | Present results of all sensitivity analyses conducted to assess the robustness of the synthesized results. | N.A. |
| Reporting biases | 21 | Present assessments of risk of bias due to missing results (arising from reporting biases) for each synthesis assessed. | Pg 8,10 |
| Certainty of evidence | 22 | Present assessments of certainty (or confidence) in the body of evidence for each outcome assessed. | Pg 8,10 |
| **DISCUSSION** | | |  |
| Discussion | 23a | Provide a general interpretation of the results in the context of other evidence. | Pg 12-15 |
|  | 23b | Discuss any limitations of the evidence included in the review. | Pg 12-15 |
|  | 23c | Discuss any limitations of the review processes used. | Pg 12-15 |
|  | 23d | Discuss implications of the results for practice, policy, and future research. | Pg 12-15 |
| **OTHER INFORMATION** | | |  |
| Registration and protocol | 24a | Provide registration information for the review, including register name and registration number, or state that the review was not registered. | Pg 4 |
|  | 24b | Indicate where the review protocol can be accessed, or state that a protocol was not prepared. | Pg 4 |
|  | 24c | Describe and explain any amendments to information provided at registration or in the protocol. | Pg 4-5 |
| Support | 25 | Describe sources of financial or non-financial support for the review, and the role of the funders or sponsors in the review. | 18 |
| Competing interests | 26 | Declare any competing interests of review authors. | 18 |
| Availability of data, code and other materials | 27 | Report which of the following are publicly available and where they can be found: template data collection forms; data extracted from included studies; data used for all analyses; analytic code; any other materials used in the review. | N/A |

**Supplementary Table 1. Ovid MEDLINE(R) ALL**1946 to April 13, 2023

|  | **Searches** | **Results** |
| --- | --- | --- |
| 1 | exp Magnetic Resonance Imaging/ | 529100 |
| 2 | (mr* adj2 (scan* or imag*)).ab,ti. | 197229 |
| 3 | (mri or Magnetic Resonance Imaging).ab,ti. | 449413 |
| 4 | 1 or 2 or 3 | 708369 |
| 5 | ((recent* or new* or first or afebrile) adj3 (epilep* or seizure*)).ab,ti. | 11231 |
| 6 | 4 and 5 | 1584 |
| 7 | exp Infant/ | 1245199 |
| 8 | (infant* or child*).ab,ti. | 1919373 |
| 9 | 7 or 8 | 2502993 |
| 10 | 6 and 9 | 571 |
| 11 | limit 10 to case reports | 163 |
| 12 | 10 not 11 | 408 |

**Supplementary Table 2. Embase Classic + Embase**1947 to 2023 April 13

| **#** | **Searches** | **Results** |
| --- | --- | --- |
| 1 | exp Magnetic Resonance Imaging/ | 1216453 |
| 2 | (mr* adj2 (scan* or imag*)).ab,ti. | 288545 |
| 3 | (mri or Magnetic Resonance Imaging).ab,ti. | 710858 |
| 4 | 1 or 2 or 3 | 1291294 |
| 5 | ((recent* or new* or first or afebrile) adj3 (epilep* or seizure*)).ab,ti. | 18757 |
| 6 | 4 and 5 | 3738 |
| 7 | exp Infant/ | 1307802 |
| 8 | (infant* or child*).ab,ti. | 2655127 |
| 9 | 7 or 8 | 3289062 |
| 10 | 6 and 9 | 1313 |
| 11 | (case adj3 report*).ab,ti. | 967914 |
| 12 | 10 not 11 | 1065 |
| 13 | (case adj2 series).ab,ti. | 150797 |
| 14 | 10 not 12 | 248 |
| 15 | 13 and 14 | 4 |
| 16 | 12 or 15 | 1069 |

**Supplementary Table 3. WOS Core Collection**, April 13, 2023

| **#** | **Search Query** | **Results** |
| --- | --- | --- |
| 1 | TI=(Magnetic Resonance Imaging OR "mri") OR AB=(Magnetic Resonance Imaging or "mri") | 516718 |
| 2 | TI=((("mr" OR "mri") NEAR/2 (scan* or imag*))) OR AB=((("mr" OR "mri") NEAR/2 (scan* or imag*))) | 207146 |
| 3 | #1 OR #2 | 549234 |
| 4 | TI=(((recent* or new* or first or afebrile) NEAR/3 (epilep* or seizure*))) OR AB=(((recent* or new* or first or afebrile) NEAR/3 (epilep* or seizure*))) | 14091 |
| 5 | #3 AND #4 | 1426 |
| 6 | (infant* or child*) (Title) OR (infant* or child*) (Abstract) | 2100791 |
| 7 | #5 AND #6 | 446 |
| 8 | (TI=((case NEAR/3 report*) NOT (Case NEAR/3 serie*)) OR AB=((case NEAR/3 report*) NOT (Case NEAR/3 serie*))) | 671388 |
| 9 | (#7) NOT #8 | 390 |

**Supplementary Table 4.** Evidence synthesis – PICO 1

| **Study ID** | **Country** | **Inclusion criteria** | **Infants** | | |
| --- | --- | --- | --- | --- | --- |
|  |  |  | **Total, N°** | **Undergoing MRI, N° (%)** | **With abnormal MRI, N° (%)** |
| **Al-Shami 2016** | QAT | Age: <14 years; First afebrile seizure | 26 (<2y) | 26 (100) | 14 (53.9) |
| **Ali 2022** | PAK | Age: 1m-18y; New-onset afebrile seizures | 83 (<1y) | N.R. | N.R. |
| **Aprahamian 2014** | USA | Age: 1m-18y; First time non-febrile seizure with focal manifestations | 72 (<18m) | N.R. | N.R. |
| **Berg 2000** | USA | Age: 1m-15y; New diagnosis of epilepsy | N.R. | N.R. | N.R. |
| **Berg 2009** | USA | Age: 1m-16y; New diagnosis of epilepsy | N.R. | 113 (N.A.) | 30 (N.A.) |
| **Cornelius 2023** | IND | Age: <12 years; New‑onset seizures (due to inherited metabolic disorder) | 32 (<1y) | N.R. | N.R. |
| **Coryell 2019** | USA | Newly diagnosed early life epilepsy; First seizures <3 year;  Established epilepsy diagnosis <42 months | N.R. | N.R. | N.R. |
| **Dirik 2018** | TUR | Age: 1-18y; New diagnosis of epilepsy | N.R. | N.R. | N.R. |
| **Eltze 2013** | GBR | Age: 1-24m; New diagnosis of epilepsy | 57 | 51 (89.5) | 37 (72.5) |
| **Gattamaneni 2022** | IND | Age: 1m-5y; New-onset seizures* | 46 | N.R. | N.R. |
| **Gowda 2019** | IND | Age: 1m-1y; First afebrile seizure | 121 | N.R. | 36 (N.A.) |
| **Hourani 2021** | LBN | Age: 6m-18y; New-onset unprovoked seizure(s) | 169 (<2y) | 169 (100) | 82 (48.5) |
| **Hsieh 2010** | USA | Age: 1-24m; New-onset afebrile seizures | 317 | 182 (57.4) | 104 (57.1) |
| **Kasap 2023** | TUR | Age: 1m-18y; First focal seizure* | 15 (<1y) | N.R. | 3 (N.A.) |
| **Stödberg 2020** | SWE | New diagnosis of epilepsy with first seizure <2y | 116 | 57 (49.1) | 37 (64.9) |
| **Trowbridge 2019** | USA | Down Syndrome + Infantile Spasms who had MRI | 36 | 36 (100) | 21 (58.3) |
| **Vecchi 2016** | ITA | Age: 1m-13y; Diagnosis of symptomatic epilepsy due to acquired and developmental etiologies and presumed symptomatic focal epilepsy | 119 (<3y) | 119 (100) | 74 (62.2) |
| ***** A proportion of the infants in this study had a febrile seizure etiology; however, the study was retained as it also includes infants within the target population of interest (first afebrile seizure or new-onset epilepsy).  **Note:** Country codes refer to the country of origin of each study, based on ISO 3166-1 alpha-3 codes. Full names: QAT = Qatar; PAK = Pakistan; USA = United States; IND = India; TUR = Türkiye; GBR = United Kingdom; LBN = Lebanon; SWE = Sweden; ITA = Italy. | | | | | |

**Supplementary Table 5.** Evidence synthesis – PICO 2

| **Study ID** | **Country** | **Inclusion criteria** | **Infants** | | | |
| --- | --- | --- | --- | --- | --- | --- |
|  |  |  | **Total, N°** | **Abnormal N.E., N° (%)** | **Abnormal EEG, N° (%)** | **Abnormal pregnancy/delivery, N° (%)** |
| **Al-Shami 2016** | QAT | Age: <14 years; First afebrile seizure | 26 (<2y) | N.R. | N.R. | N.R. |
| **Ali 2022** | PAK | Age: 1m-18y;  New-onset afebrile seizures | 83 (<1y) | N.R. | N.R. | N.R. |
| **Aprahamian 2014** | USA | Age: 1m-18y;  First time non-febrile seizure with focal clinical manifestations | 72 (<18m) | N.R. | N.R. | N.R. |
| **Berg 2000** | USA | Age: 1m-15y; New diagnosis of epilepsy | N.R. | N.R. | N.R. | N.R. |
| **Berg 2009** | USA | Age: 1m-16y;  New diagnosis of epilepsy | N.R. | N.R. | N.R. | N.R. |
| **Cornelius 2023** | IND | Age: <12 years; New‑onset seizures (due to inherited metabolic disorder) | 32 (<1y) | N.R. | N.R. | N.R. |
| **Coryell 2019** | USA | Newly diagnosed early life epilepsy; First seizures <3 year;  Established epilepsy diagnosis <42 months | N.R. | N.R. | N.R. | N.R. |
| **Dirik 2018** | TUR | Age: 1-18y; New diagnosis of epilepsy | N.R. | N.R. | N.R. | N.R. |
| **Eltze 2013** | GBR | Age: 1-24m;  New diagnosis of epilepsy | 57 | 25 (43.9) | N.R. | N.R. |
| **Gattamaneni 2022** | IND | Age: 1m-5y; New-onset seizures | 46 | N.R. | N.R. | N.R. |
| **Gowda 2019** | IND | Age: 1m-1y; First afebrile seizure | 121 | 85* (70.2) | N.R. | 46^#^ (38.0) |
| **Hourani 2021** | LBN | Age: 6m-18y;  Diagnosis of ≥1 unprovoked seizure | 169 (<2y) | 136* (80.5) | N.R. | N.R. |
| **Hsieh 2010** | USA | Age: 1-24m;  New-onset afebrile seizures | 317 | 34 (10.7) | 105^£^ (33.1) | 30^$^ (9.5) |
| **Kasap 2023** | TUR | Age: 1m-18y; First focal seizure | 15 | N.R. | N.R. | N.R. |
| **Stödberg 2020** | SWE | New diagnosis of epilepsy with first seizure <2y | 116 | 46* (39.7) | 73 (62.9%) | 18^$^ (15.5) |
| **Trowbridge 2019** | USA | Down Syndrome + Infantile Spasms who had MRI | 36 | 36 (100) | 30 (83.3) | N.R. |
| **Vecchi 2016** | ITA | Age: 1m-13y; Diagnosis of symptomatic epilepsy due to acquired and developmental etiologies and presumed symptomatic focal epilepsy | 119 (<3y) | 42 (35.3) | 110 (95.7) | N.R. |

***** Cases reporting evidence of developmental delay at neurological assessment were included under the category of abnormal neurological examination.

**^#^** A significant history of perinatal insult was included under the category of abnormal pregnancy/delivery.

^$^ Low birthweight and preterm birth were included under the category of abnormal pregnancy/delivery.

^£^ EEG was performed in 90.3% of the sample; the reported percentage of abnormal findings refers to this subset.

**Note:** Country codes refer to the country of origin of each study, based on ISO 3166-1 alpha-3 codes. Full names: QAT = Qatar; PAK = Pakistan; USA = United States; IND = India; TUR = Türkiye; GBR = United Kingdom; LBN = Lebanon; SWE = Sweden; ITA = Italy.

**Supplementary Table 6 – Risk of Bias assessment**

|  | **Inclusion criteria** | **Subjects and setting** | **Exposure measure** | **Condition criteria** | **Confounding factors identified** | **Confounding factors dealt** | **Outcome measurement** | **Statistical analysis** |
| --- | --- | --- | --- | --- | --- | --- | --- | --- |
| **Ali 2022** | Yes | Yes | Unclear | Yes | Yes | Yes | Unclear | Unclear |
| **Al-Shami 2016** | Yes | No | Unclear | Yes | No | No | Unclear | Yes |
| **Aprahamian 2014** | Yes | Yes | Unclear | Yes | Unclear | Unclear | Yes | Unclear |
| **Berg 2000** | Yes | Yes | Yes | Yes | Yes | Yes | Yes | Yes |
| **Berg 2009** | Yes | Yes | Unclear | Yes | Yes | Yes | Unclear | Unclear |
| **Cornelius 2023** | Yes | Yes | Unclear | Yes | Unclear | No | Not applicable | Yes |
| **Coryell 2019** | Yes | Yes | Yes | Yes | Unclear | Unclear | Yes | Yes |
| **Dirik 2018** | Yes | No | Yes | Yes | No | No | Yes | Unclear |
| **Eltze 2013** | Yes | Yes | Unclear | Yes | Yes | Yes | Yes | Yes |
| **Gattamaneni 2022** | Yes | No | Unclear | Yes | No | No | Yes | Yes |
| **Gowda 2019** | Yes | No | Unclear | Yes | No | No | Yes | Unclear |
| **Hourani 2021** | Yes | Yes | Yes | Yes | Yes | Yes | Yes | Yes |
| **Hsieh 2010** | Yes | Unclear | Yes | Yes | Unclear | Unclear | Not applicable | Yes |
| **Kasap 2023** | Yes | Yes | Unclear | Yes | Yes | Unclear | No | Yes |
| **Stödberg 2020** | Yes | Yes | Unclear | Yes | Yes | Yes | Yes | Yes |
| **Trowbridge 2019** | Yes | Unclear | Yes | Yes | Yes | Unclear | Yes | Yes |
| **Vecchi 2016** | Yes | Yes | Unclear | Yes | Yes | Yes | Yes | Yes |

**Note**: Risk of bias was assessed using the JBI Critical Appraisal Checklist for Analytical Cross-Sectional Studies (<https://jbi.global/sites/default/files/2020-08/Checklist_for_Analytical_Cross_Sectional_Studies.pdf>)

**Question:** What is the effectiveness of brain MRI performed in both emergency and non-urgent settings in identifying the underlying cause of seizures in infants with a first afebrile seizure or new-onset epilepsy?

**Setting:** Emergency and non-urgent settings

**Supplementary Table 7.** Summary of Findings for PICO 1 (GRADE assessment)

| **Certainty assessment** | | | | | | | **Impact** | **Certainty** | **Importance** |
| --- | --- | --- | --- | --- | --- | --- | --- | --- | --- |
| **№ of studies** | **Study design** | **Risk of bias** | **Inconsistency** | **Indirectness** | **Imprecision** | **Other considerations** |  |  |  |
| **Diagnostic yeld** | | | | | | | | | |
| 17 | non-randomised studies | not serious | not serious | very serious^a^ | serious^b^ | none | - 17 studies were analyzed, including 1209 infants who met the inclusion criteria. - 753 infants underwent brain MRI, with the percentage of MRI utilization ranging from 49.1% (*Stödberg 2020*) to 100.0% (*Al-Shami 2016, Hourani 2021, Trowbridge 2019, Vecchi 2016*). - MRI abnormalities were identified in 438 infants, with the percentage of abnormal findings ranging from 48.5% (*Hourani 2021*) to 72.5% (Eltze 2013). - 4 studies did not report the total number of infants evaluated (*Berg 2000, Berg 2009, Coryell 2019, Dirik 2018*), leading to potential underestimation of the true sample size. - 9 studies lacked explicit data on the number of infants who underwent MRI (*Ali 2022, Aprahamian 2014, Berg 2000, Cornelius 2023, Coryell 2019, Dirik 2018, Gattamaneni 2022, Gowda 2019, Kasap 2023*), preventing precise calculation of MRI utilization rates in those cases. - The reporting of MRI abnormalities was also incomplete, with 8 studies failing to specify the number of abnormal findings in infants (*Ali 2022, Aprahamian 2014, Berg 2000, Cornelius 2023, Coryell 2019, Dirik 2018, Gattamaneni 2022, Kasap 2023*). | ⨁◯◯◯ Very low^a,b^ | 9 |

#### Explanations

a. Variability in MRI protocols, inclusion criteria, and the classification of abnormalities limits the direct applicability of findings to all clinical settings. Some studies do not distinguish between clinically significant and incidental findings.

b. Confidence in effect estimates is limited by incomplete data reporting, wide variability in diagnostic yield across studies, and differences in neuroradiologists’ expertise. While some findings are based on small sample sizes, substantial variability is also observed within these cohorts.

**Question:** Which clinical features in infants with a first afebrile seizure or new-onset epilepsy are associated with MRI-detected abnormalities that explain the cause of seizures?

**Setting:** Emergency and non-urgent settings

**Supplementary Table 8.** Summary of Findings for PICO 2 (GRADE Assessment)

| **Certainty assessment** | | | | | | | **Impact** | **Certainty** | **Importance** |
| --- | --- | --- | --- | --- | --- | --- | --- | --- | --- |
| **№ of studies** | **Study design** | **Risk of bias** | **Inconsistency** | **Indirectness** | **Imprecision** | **Other considerations** |  |  |  |
| **Clinical predictors** | | | | | | | | | |
| 17 | non-randomised studies | serious^a^ | not serious | very serious^b^ | serious^c^ | none | - 17 studies were analyzed, including 1209 infants who met the inclusion criteria. - 404 infants had abnormal neurological examinations, with the percentage ranging from 10.7% (*Hsieh 2010*) to 100.0% (*Trowbridge 2019*). - 318 infants had abnormal EEG findings, with the percentage ranging from 33.1% (*Hsieh 2010*) to 95.7% (*Vecchi 2016*). - 94 infants had abnormalities related to pregnancy or delivery, with the percentage ranging from 9.5% (*Hsieh 2010*) to 38.0% (*Gowda 2019*). - 4 studies did not report the total number of infants evaluated (*Berg 2000, Berg 2009, Coryell 2019, Dirik 2018*), potentially leading to an underestimation of the sample size. - 10 studies lacked explicit data on abnormal neurological examinations (*Al-Shami 2016, Ali 2022, Aprahamian 2014, Berg 2000, Berg 2009, Cornelius 2023, Coryell 2019, Dirik 2018, Gattamaneni 2022, Kasap 2023*) - 13 studies did not provide data on abnormal EEG findings (*Al-Shami 2016, Ali 2022, Aprahamian 2014, Berg 2000, Berg 2009, Cornelius 2023, Coryell 2019, Dirik 2018, Eltze 2013, Gattamaneni 2022, Gowda 2019, Hourani 2021, Kasap 2023*) - 14 studies failed to report abnormalities related to pregnancy or delivery (*Al-Shami 2016, Ali 2022, Aprahamian 2014, Berg 2000, Berg 2009, Cornelius 2023, Coryell 2019, Dirik 2018, Eltze 2013, Gattamaneni 2022, Hourani 2021, Kasap 2023, Trowbridge 2019, Vecchi 2016*). | ⨁◯◯◯ Very low^a,b,c^ | CRITICAL |

#### Explanations

a. Many studies lack complete reporting on the total number of infants evaluated, the presence of abnormal neurological examinations, EEG findings, and perinatal complications. This increases the risk of selection and reporting bias, potentially skewing the associations between clinical features and MRI abnormalities.

b. Variability in the definitions of clinical features (e.g., focal vs. generalized seizures, abnormal neurological exams), inconsistencies in EEG interpretations and abnormal neurological examination reporting, and the lack of standardized population descriptions limit the direct applicability of findings. Additionally, some studies do not account for confounders that may influence MRI findings, further reducing the strength of the associations observed.

c. The limited sample sizes, along with missing, non-standardized data on clinical features and reporting inconsistencies, reduce confidence in the precision of effect estimates.
